# Dr. J. Hall Moore's Address

**Published:** 1879-03

**Authors:** J. Hall Moore

**Affiliations:** PRESIDENT.


### ARTICLE II.

### *Address before the Virginia State Dental Association.*

BY DR. J. HALL MOORE, PRESIDENT.

Delivered December 10th, 1878.

*Gentlemen of the Virginia State Dental Association.*—It is now nine years since the organization of our Association. Each year our meetings have been marked by increased interest and fuller attendance, so we may now congratulate each other that the Virginia State Dental Association is no longer a mere experiment but an accomplished fact; and it only remains with ourselves to make it a power, not only in the profession, but in the community and the State.

Our former meetings have attracted a large share of attention and interest from the people and press, notwithstanding they have been held at a season when the National and State Legislatures were in session, and the discussion of questions of grave public moment by those bodies was diverting the minds of the people from the consideration of minor matters. Already the question has been frequently asked, "when does your State Dental Society meet?"

I congratulate you that at a time like this, at the close of a year almost unprecedented for general depression of business, and the financial pressure which has borne so heavily upon all classes of the community, so many of you have found it in your power to leave your homes and business to assemble here in the Capital City of our beloved State, to consult together upon matters relating to our specialty.

I congratulate you, gentlemen, that so far as we are advised, no breach has been made in the ranks of our Association during the past year by the hand of death. While the King of terrors has been reaping a heavy harvest in some of our sister States, and many have fallen at the post of duty, and others have seen their homes desolated by the snatching away of their loved ones, we have been mercifully spared such a visitation.

With thankful hearts to the Giver of all good who has been thus merciful and kind to us, let us pause a moment and drop a tear of sorrow for those who have fallen, and lift a prayer for the stricken ones in their desolation.

The first great object of our meeting, is to communicate to each other what we have been able to glean from those sources of knowledge which each may have found open to him, and particularly those points which experience and thought have developed, and which as we love our profession and desire its advancement, we would not keep to ourselves, but desire that every brother practitioner should know and use for the public good.

The other great object before us, is to confer with regard to the best means of cultivating a spirit of good feeling and fraternity among the members of our profession; of best promoting its interests, and of elevating it to that place which it deserves and to which it is justly entitled. Whatever others may *think*, we *know* that our profession is a noble one. The great end and aim of every true practitioner of dentistry is the relief of human suffering. It is true, that like other men, we and our families have natural wants and necessities which must be supplied; therefore we are compelled to seek some remuneration for our services, and it is only right and just that we should do so; but the man who makes pecuniary profit and personal wealth the goal of his ambition, and is actuated in the practice of his profession by personal considerations only, is not fit for so high a calling as that of the dentist and should seek some other walk in life where there is less demand for self-sacrifice, and love for all humanity.

Next to the general practice of medicine, our mother profession, there is no calling which demands so much self-abnegation and so large a degree of time sympathy and kindly feeling. The true dentist should be literally full to overflowing with the "milk of human kindness."

He has constantly to deal with physical suffering in its acutest form. To relieve pain, he is often compelled to inflict, for the moment, a much greater degree of agony. To prevent and provide against future suffering, he is frequently obliged to inflict an amount of torture from which the stoutest heart turns with horror.

We have all of us seen strong men who had faced the cannon's mouth, and advanced to the deadly charge without the slighest thought of fear, blanch and turn cold at the sight of a nerve broach or a pair of forceps; and who feared a dentist's chair, more than the bursting shell or whizzing minie ball.. By far the larger number of those who come under our hands for treatment, are the weak, nervous and timid, who demand and are entitled to our fullest sympathies and tenderest services. Many of them come to us worn down with pain and suffering, and in such condition as to appeal at once to all the sympathies of our nature.

To be successful in the highest sense, the dentist must be firm as well as gentle. He must have a strong hand, well trained to use its strength with the most delicate touch. He must have a kind, sympathizing heart; he must know just when to manifest his sympathy for his suffering patient, and when to steel himself into an appearance of indifference. He must be patient, industrious, untiring; charitable to the faults and frailties of humanity, not easily offended, urbane, cleanly and neat in person and surroundings, self-possessed, punctual, earnest and laborious. He should be ready when the work of the day is over to light the students lamp and carefully peruse what other men have observed and written down for his instruction, or write out the result of his own observations and experience, that others may profit thereby in the great work of mitigating human suffering.

The time was, when men who professed to practice dentistry kept secret from others their peculiar methods of operating, that they alone might derive profit and emolument therefrom. I have myself paid as high as a hundred dollars for three hours instruction in some new method, and there are several gentlemen now present who will bear witness that a large part of our dental education was obtained in that way. But I am happy to say that day has long since passed away, and that now all true minded dentists are ready to communicate whatever discoveries they may make for the benefit of the profession, as the well filled pages of nearly a dozen dental journals, published in the United States alone, will testify.

Our profession has made great and rapid strides in the last quarter of a century. Some of the gentlemen now before me can recollect when a dental college was not only unheard of, but undreamed of. We remember when the name dentist was rather one of reproach, regarded as almost synonymous with quack or humbug. The few honest, earnest men who practised dentistry from right and true motives had to bear the reproach and dishonor of being associated in the minds of the public with the rough barber tooth drawer, who cared only for the quarter or half dollar which he received from his victim, and who, in the majority of cases could not tell an incisor from a molar.

When a few far seeing, earnest men began to discern the public need for educated dentists, and proposed to found a college for the instruction and training of those who desired to become such, they were met with sneers and division. Notwithstanding the great, and what seemed to many insurmountable difficulties in the way, the Baltimore Dental College was opened, and at once found men of the right stamp ready to enter its halls, thankful that the opportunity was afforded them to prepare themselves properly for the discharge of the duties of their chosen profession.

Some of these men have been an honor not only to their calling, but to the country, and have aided greatly in the work of bringing our profession up to its present high state of dignity and usefulness. We are happy to know that some of them are still spared, and are in the harness, working to elevate their chosen profession to a still higher standard. Now, instead of our dental college, we have no less than ten, in the United States, and some of the principal medical schools have been glad to acknowledge dentistry as a specialty of medicine, and to appoint men qualified to teach it in their colleges. Not only in this country has the importance of dental education been recognized, but in several of the States of Europe Dental Colleges have been founded.

Within the past year the Government of Great Britian has awaked to the importance of proper training for the practice of dentistry, and by legal enactment made it a specialty of medicine. By act of Parliament those who shall hereafter engage in practising this specialty must have a regular medical education, and in addition thereto be able to pass a most rigid examination as to their qualifica tions as dentists. The English dentist of the future will be as well grounded in Anatomy, Physiology, Pathology, Chemistry, *Materia Medica* and Surgery as the general medical practitioner. The student who can pass the examination required by the "Dental Examining Board of the Royal College of Surgeons of England" for the degree of Dental Surgeon, will be well fitted to practise either medicine or dentistry, and will have no occasion to be ashamed of his choice of dentistry as a profession.

While much has been accomplished in the last few years, much remains to be done. As we all know too well, there are many men, even in this enlightened age, calling themselves dentists, who are in every respect unworthy of the name without the first qualification except impudence, if that may be called a qualification. They palm themselves off upon a too easily gulled community, by flaming advertisements in which they do not hesitate to claim superiority over those who have labored earnestly and honestly to fit themselves for their work. By offering to work at prices little above common laborers wages, they deceive the public, unfortunately not yet sufficiently educated upon the subject to estimate rightly the value of dental operations; they sacrifice thousands of teeth annually and bring dishonor upon the fair name of an honorable profession. The deluded and swindled victims of these pretenders are not the only sufferers by this state of affairs.

People generally do not discriminate between the educated, honest man who tries to serve his patients well and faithfully, and to minister to the good of his fellow men, and the dishonest quack. Therefore the profession as such, is held responsible for all the injury done by the ignorant pretenders, much of the responsibility of this State of affairs lies at our door. We have not done our duty in the premises, and we are not doing what we ought now, in the way of enlightening the public mind as to the difference between the qualified dentist and the mere charlatan.

In many of our Dental Societies the lines have not been drawn rigidly enough. The desire to make a large show of numbers has led to great laxity in receiving members into these societies; and thus many men, wholly unworthy have been admitted into membership, and in many instances allowed to take prominent part in them, and use them for their own sinister ends.

Again, in the great desire to secure practice, some who know better, and who, as regards educational qualifications and manipulative skill, are worthy to take high rank, so far forget what is due to themselves, their professional brethren, and an honorable profession, as to resort to the dishonorable tricks of the quack and mountebank to bring themselves before the public eye for exclusively selfish purposes.

Some of our Dental Colleges too, in the race for popularity, and fearing to give offence to their patrons, graduate and send our young men to practise, whom they know to be utterly unqualified and in every way unfitted by nature as well as by lack of proper education for the discharge of those functions which belong to the dentist. Thus the professional standard is lowered, and the profession itself brought into disrepute.

Gentlemen, these things ought not to be, and the remedy lies with us. The public mind is to be educated, and the honorable high minded dentist alone can do it.

We should teach those who come under our influence to discriminate between the quack, (even if he holds a college diplomacy,) and the honest man who loves his profession, and practises it for noble ends, and not merely to make money.

We should rigidly exclude from our fellowship *all* who resort to unworthy means of any kind to break down their fellow practitioners, or build themselves up. While we should be extremely careful to avoid everything which could possibly be used to the disadvantage of a time professional brother, and exercise the greatest charity towards the failings of those who are honest, by endeavoring to do right, we should not hesitate to denounce in unmeasured terms those whom we know to be mere pretenders.

We should be animated by a more kindly spirit towards each other. Jealousy and envy are wholly unworthy of men who are engaged in so honorable a work as we know ours to be.

We who are older should counsel our younger brethren, and do all that we can to encourage them, to pursue their calling in an honorable manner for honorable ends. We should endeavor to stimulate their ambition to excel in everything that relates to their profession and do all in their power to ennoble it. This we should do not only by precept but by example. If we have opportunity to see when a brother has failed in the endeavors to perform some operation which has been entrusted to him, we should go to him, show him how the failure was caused and how it might have been prevented so that he may be able to guard against the same error in the future.

Those whose lot it is to practise in the same community should cultivate a sentiment of friendship for each other, and avoid everything like professional jealousy. They should meet as often as possible and confer as to the best modes of practice, and do all in their power to advance each other's interest, giving their patients and the public to understand that they are friends, and not rivals, brothers in an honorable calling, and caring more for the honor of their profession than for personal aggrandizment.

If there be any rivalry let it be a generous one to excel in all that is ennobling, and in advancing the great end which should be nearest the heart of each; the relief of human suffering. There is no reason whatever why two or more men practising such a profession as ours, in the same town or city, should not be the best of friends, and it would be so if we were all actuated by right and pure motives.

I trust that the present session may be as pleasant and harmonious as those which have preceded it. That those who are with us for the first time will be encouraged to take hold and labor in the good cause, and that we will all determine that whatever may have been our course in time past, from this time onward we will do all in our power to elevate our profession, and avoid everything which can by any possibility bring it into reproach or dishonor.

